# Sensitive electrochemical detection of total sugars in food using NiFe alloy nanowires

**DOI:** 10.1007/s00604-025-07663-3

**Published:** 2025-11-17

**Authors:** Bernardo Patella, Nadia Moukri, Francesca Mazzara, Sonia Carbone, Roberto Luigi Oliveri, Giuseppe Aiello, Michele Russo, Claudia Torino, Antonio Vilasi, Vuslat Buk Juska, Alan O’Riordan, Rosalinda Inguanta

**Affiliations:** 1 Dipartimento di Ingegneria, Università degli Studi di Palermo, Viale delle Scienze, Palermo, 90128 Italy; 2 Dipietro Group, Melilli, 96010 Italy; 3 Institute of Clinical Physiology, National Research Council, Reggio Calabria, Reggio Calabria, 89124 Italy; 4 Precision Electrochemical Nanosensor Group, Tyndall National Institute, University College Cork, Cork, T12 R5CP Ireland

**Keywords:** Sugar determination, Electrochemical sensors, Nanowires, NiFe alloy, Food analysis

## Abstract

An innovative electrochemical sensor is introduced based on a nickel-iron (NiFe) alloy, designed for rapid, cost-effective, and straightforward sugar determination in food. The sensor features vertically aligned NiFe nanowire arrays, providing a high surface area for enhanced performance. Sensor calibration was performed for various mono- and disaccharide reducing sugars. In the linear range 0.05–0.3 mM, sensors exhibited a sensitivity of 0.642 µA µM⁻¹·cm⁻², with a detection limit of 2.57 µM for monosaccharides and a limit of quantification of 14 µM. In the same linear range, for disaccharides a sensitivity of 0.355 µA µM⁻¹·cm⁻², a detection limit of 4.62 µM and a limit of quantification of 14 µM were found. The sensors displayed excellent selectivity towards reducing sugars with negligible interference from glycerin, citric acid, Cu^2+^, K^+^, Na^+^, Cl^−^, Ca^2+^, NO_3_^−^, Mg^2+^, SO_4_^2−^, uric acid, ascorbic acid, gallic acid and ethanol. Non-reducing sugars are also detectable following a simple sample pre-treatment step. Sensors were validated by measuring sugar concentrations in real samples (honey, fruit, milk, coke, juices, etc.), yielding recoveries above 96% thereby demonstrating robust selectivity against diverse interferents. These results were corroborated by conventional analysis techniques, showing excellent correlation. The proposed non-enzymatic nanowire sensor presents remarkable advantages, including exceptional stability and cost-effectiveness. It enables ultra-fast sugar detection in complex matrices, making it a powerful tool for food quality control and industrial process monitoring. This study report, for the first time, the effective application of NiFe nanowire arrays for total sugar quantification in food matrices. The developed sensors show outstanding performance compared with both enzymatic and non-enzymatic systems.

**Graphical abstract:**

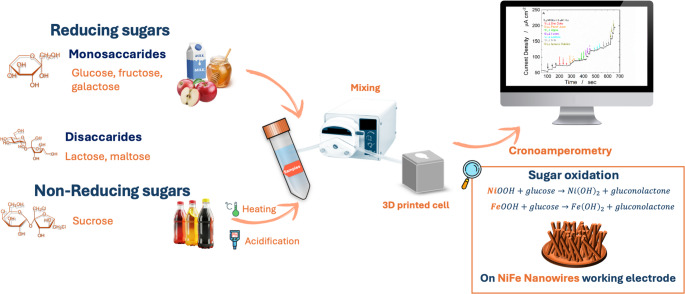

**Supplementary Information:**

The online version contains supplementary material available at 10.1007/s00604-025-07663-3.

## Introduction

Sugars can be categorized as reducing or non-reducing. Reducing sugars (RSs), like glucose and fructose, are a class of sugars that have either a free aldehydic (aldose sugars) or ketonic group (ketose sugars). These functional groups allow them to act as a reducing agent under specific conditions. RSs play many important roles in, for example, clinical diagnostics [1], the food industry [2], and electrochemical devices [3]. Quantification of RSs in foodstuffs is essential for various purposes, such as for nutritional evaluation, adulteration detection, and quality control during food processing. Amongst all of the RSs, glucose has attracted the most interest. Monitoring of glucose is very important for the management of diabetes and related diseases, and thus is of great interest to healthcare professionals and society in general [4]. RSs quantification in the agricultural, food, and beverage industry is very important as sugars serve as indicators for product characteristics and authenticity and contribute to qualities such as taste and aroma [5, 6]. For example, in the production of ethanol from sugarcane, the quantification of total RSs allows the evaluation of the quality of the raw material and thus optimization of the production chain [7]. The amount of RSs plays a key role in the storage of potatoes [8] and rice [9] and in evaluating milk stability during the milk heating process [10]. The classification of adulteration of honey [11] is made based on its RSs composition. Total RSs quantification in wines allows the evaluation of various parameters such as the alcohol content, the end of fermentation, and, in general, tracking the production process [12]. Furthermore, the quantification of total RSs in foodstuffs is also essential to comply with legal requirements [13]. Many foods contain both natural and added sugars, and the body cannot distinguish between them as they are chemically identical [14]. Consuming foods high in added sugars is concerning as they are highly calorific and contribute to weight gain and various health issues [15]. Therefore, accurately quantifying sugars in foods is essential for human health [16]. Several lab-based analytical techniques are employed for their quantification, such as high-performance liquid chromatography [6], as well as colorimetric [17] and spectrophotometric methods [18]. Titration methods based on Fehling’s reagent (official method of analysis of the Official Association of Analytical Chemists, method 923.09) [19] or iodometry [20] are also widely used. All these methods, however, are laborious, complicated, and require specialized personnel, reagents and equipment that do not allow continuous and/or real-time analysis [12]. To overcome these issues, the development of RSs electrochemical sensors becomes particularly important [21]. For sugar detection, both enzymatic [22] and non-enzymatic [23] electrochemical sensors (ESs) have been developed. Non-enzymatic sensors, based on the direct oxidation of sugars on an electrode surface, are very promising because they can be easily stored and have very fast response times [21]. Electrochemical sensors have small dimensions, are economical, portable can be used with low energy consumption [24, 25]. In addition, these sensors, coupled with nanostructured materials, exhibit very low Limit of Detection (LOD) values due to their very high surface area [26–30]. Noble metals (platinum, gold) with different nanostructured shapes (nanowires, nanotubes, nanoflowers, nanoparticles) have been used to build electrochemical sensors [31–34] for non-enzymatic sugar detection. However, these metals are extremely expensive and susceptible to interference by chloride ions [1]. Cheaper materials for use as non-enzymatic sugar sensors include less noble metals such as nickel, copper and cobalt [35–37]. Recent studies have highlighted the crucial role of nanostructured and multimetallic surfaces in enhancing electrochemical sensitivity and selectivity, while also demonstranting the versatility of nanomaterials in both sensing and biomedical applications [38]. For example, electrochemical nano-imprinting of trimetallic dendritic surfaces has been demonstrated to provide a highly active and reproducible platform for antibiotic detection [39]. Comparative electrochemical studies between phyto-fabricated and chemically synthesized silver nanoparticles revealed distinct redox behaviors toward hydrogen peroxide detection, underlining the effect of the synthesis routes on performance [40]. A body of work in the literature has demonstrated that Ni alloys (NiFe, NiCu, NiCo, etc.) and Ni-metalloid alloys (NiB, NiP, etc.), are very suitable materials for non-enzymatic sugar detection because they exhibit better catalytic performance than individual metals. Monometallic systems (Ni or Fe) often suffer from limited catalytic stability or slow electron transfer, whereas bimetallic NiFe alloys benefit from a synergistic effect that enhances catalytic efficiency and stability [41–43]. Non-enzymatic glucose detection was demonstrated by Song et al. using a NiFe layered double hydroxide nanosheet-based sensor [44]. This material exhibited high sensitivity (6.61 µA·µM^− 1^·cm^− 2^) in a wide linear dynamic range (0.01–1.01 mM), with a limit of detection (LOD) of 3 µM. Similarly, a NiFe-polyaniline hybrid electrode, synthesized by Lakhdari et al. [45], exhibited glucose detection in the linear range from 10 µM to 1 mM with a good sensitivity (1.05 µA µM^− 1^·cm^− 2^) and a low LOD of 0.5 µM. Similarly, Pan et al., demonstrated a NiCu bimetallic metallorganic framework-based glucose sensor with validation in human serum samples with excellent recovery rates [46]. As demonstrated in [47], bismuth film doped with Ni and Co showed high catalytic activity for glucose oxidation. A dynamic range of 1 µM to 3.5 mM, a sensitivity of 0.677 (doping with Ni) and 2.32 (doping with Co) mA µM^− 1^·cm^− 2^, and a LOD of 1 µM (doping with Ni) and 4 µM (doping with Co) was demonstrated. The approach followed by Wei et al. is interesting in that, through electrodeposition, they obtained dendritic core-shell copper-nickel alloy@metal oxide for efficient glucose detection [48]. Very recently, a sensor based on FeCoNiMnCr high entropy alloys has been shown by Gokul et al. [49]. Nevertheless, the reported materials still suffer from several drawbacks, including low sensitivity, high cost, limited stability, and the ability to quantify only glucose rather than all reducing sugars.

In this work, an array of ordered NiFe alloy nanowires (NWs), synthesized via template-assisted electrodeposition, was employed as the active material for the electrochemical detection of total reducing sugars (glucose, fructose, galactose, lactose, and maltose) in food samples. NiFe-based materials have recently attracted considerable attention for electrochemical sensing owing to their excellent catalytic and electronic properties. The synergistic interaction between Ni and Fe enhances the redox activity of the Ni²⁺/Ni³⁺ and Fe²⁺/Fe³⁺ couples, facilitating electron transfer and improving catalytic kinetics compared to monometallic counterparts [50, 51]. Moreover, the nanowire morphology provides a highly accessible electroactive surface area and ensures rapid electron diffusion along the one-dimensional structure [52]. These combined features make NiFe alloy nanowires an excellent platform for the sensitive and reliable quantification of total RSs in complex food matrices. Although the NiFe alloy has been previously studied for glucose detection [44, 45], no prior work has utilized an array of NiFe nanowires (NWs) in an electrochemical sensor for sugar analysis. The results, when compared with existing non-enzymatic electrochemical sensors, show that the NiFe NW-based sensor developed in this study enables sugar quantification across a wide concentration range and, notably, achieves exceptionally high sensitivity. This is a significant advancement beyond the state of the art, as enhanced sensitivity directly contributes to improved accuracy and precision in analytical applications. The main limitation of the reported NiFe-nanostructured systems is the difficulty in achieving controlled nanostructures and reproducible synthesis. Our nanowire array fabrication approach addresses this by ensuring uniform architectures and enhanced electron pathways. A further key innovation of this work lies in the expanded scope of detection. While most literature reports focus solely on glucose, only few studies have addressed the electrochemical quantification of total reducing sugars [5, 8, 21, 53, 54]. Here, we present a systematic investigation encompassing different mono- and disaccharides, demonstrating the sensor ability to detect and quantify all of them effectively. The quantification of monosaccharides and disaccharides occurs in the linear range from 0.05 to 0.3 mM, with a sensitivity of 0.642 µA µM⁻¹·cm⁻² and 0.355 µA, respectively, while the LOD was 2.57 and 4.62 µM, respectively. This capability positions the proposed sensor as a robust and versatile platform for total sugar analysis. Another particularly novel aspect of this study is the detection of non-reducing sugars, which was achieved through a simple pre-treatment of the sample, an approach rarely explored in prior research. Finally, the sensor was successfully applied to the analysis of various real food samples, including honey, peach juice, regular and diet coke, apple extract, milk, and an isotonic solution, further validating its practical applicability in complex matrices.

## Experimental

### Chemicals and reagents

Sodium Chloride, Calcium Chloride, Magnesium sulphate, Potassium chloride, Copper sulphate, citric acid, glycerin, uric acid, gallic acid, ascorbic acid, ethanol, glucose, fructose, lactose, galactose, maltose, sucrose, nickel sulphate, nickel chloride, boric acid, iron sulphate, chloroform, sodium hydroxide, potassium hydroxide, sulphuric acid, KNaC_4_H_4_O_6_.4H_2_O, methylene blue, were purchased from Sigma Aldrich with analytical grade and used as received. All solutions were prepared with deionized water (DI, resistivity of 18 MΩ cm^− 1^). Nanoporous polycarbonate membrane (Whatman^®^, Cyclopore) was purchased from Cytiva and used as received.

### Electrode fabrication and characterization

Nanostructured electrodes made from NiFe alloys were synthesized using a template electrosynthesis method consisting of a two-step process. First, a thin layer of gold was sputtered onto one surface of a polycarbonate membrane template to render it electrically conductive. Onto this surface, a Ni layer was then electrodeposited using potentiostatic deposition, using a platinum mesh as counter electrode, at −1.5 V (versus standard calomel electrode (SCE)) for 90 min in a Watt’s bath (300 g/L NiSO_4_·6 H_2_O, 45 g/L NiCl_2_, 45 g/L H_3_BO_3_) [55]. This Ni layer acted as both a mechanical support and a current collector for the nanostructures. The deposition solution used for nanowire (NWs) synthesis was a Watt’s bath modified with FeSO_4_•7H_2_O. The electrodeposition of NWs was performed using a pulsed potential, switching between − 0.65 V and − 1.35 V versus SCE for 100 cycles. Following NW synthesis, polycarbonate membranes were dissolved in chloroform at room temperature. To ensure complete removal of the template, the process was repeated four times (5 min each) using fresh solvent for each step. Figure S1 shows a scheme of the fabrication process of the NWs-based electrodes. To evaluate the crystalline structure of the resulting alloy, electrodes were characterized by X-ray diffraction (XRD) using a RIGAKU diffractometer (model: D-MAX 25600 HK). Morphology and elemental composition were analyzed using scanning electron microscopy (SEM) with a FEI FEG-ESEM (model QUANTA 200) equipped with an X-ray Energy Dispersive probe (EDS). Details of the characterization methods can be found in our previous reports [55].

### Electrochemical detection of RS

The electrodeposited NiFe NWs served as sensing material for sensitive and accurate reducing sugar detection in alkaline solution (NaOH 0.1 M, pH 12). An alkaline solution was chosen since it has been shown that the oxidation of sugars occurs more efficiently at high pHs, while in a neutral solution, a non-linear response was observed [56, 57]. Glucose, fructose, galactose, lactose, and maltose were selected as models for reducing sugar, while sucrose was selected as a model non-reducing sugar. Electrochemical tests were conducted using a 3D-printed cell (maximum sample volume of 4 mL, while the geometric area of a working electrode was 0.785 cm²) equipped with microfluidic channels and a peristaltic pump to continuously stir the solution. The microfluidic channels have a diameter of 1 mm and pass through the cell, enabling the mixing of the solution (Figure S2). Measurement of reducing sugars was conducted while stirring. A platinum wire and a saturated calomel electrode (SCE) were employed as counter and reference electrodes, respectively. The impact of scan rate on glucose detection was investigated within a range of 5–500 mV s^− 1^ using 5 mM glucose in 0.1 M NaOH solution. To calculate the double-layer capacitance (C_dL_), CVs were performed at different scan rates (10–50 mV s^− 1^) in 0.1 M NaOH solution within a potential range devoid of faradaic processes. Electrochemical detection of reducing sugars was performed using chronoamperometry at + 0.5 V versus SCE. All experiments were repeated five times, and the average values were plotted in the calibration curve. Sugar detection was also carried out in the presence of interfering species such as chloride, sodium, and potassium ions, as well as uric, citric, and lactic acids. In addition, natural antioxidant species present in the food matrix, such as ascorbic and gallic acid, were also tested as interferents. The stability of the NiFe electrode was established by recalibrating (for glucose) every week for a month. Between calibrations, the electrode was stored in air and at room temperature.

To validate the sensor performance with real samples, a commercial 5% isotonic glucose solution, honey, milk, regular and diet coke, and fruit juices were diluted with 0.1 M NaOH and tested without any additional pre-treatment. For honey, a specific amount was weighed and dissolved in 0.1 M NaOH. For apple juice, a sample of an apple was weighed, shredded, and dissolved in deionized (DI) water. Mixtures were then subjected to ultrasound treatment for five minutes. The quantification of RSs in real samples was carried out by measuring the change in current density upon the addittion of a known aliquot of the sample. The measured value was then divided by the corresponding sensitivity (mono or disaccharide) to determine the RSs concentration. For samples where non-reducing sugars could also be detected (i.e., coke, diet coke, juice, and apple), the total reducing sugar concentration was estimated following acidification with 1 M HCl and heating at 60 °C for 1 h. The recovery (%) was calculated using the following equation [58]:

Recovery (%)= (1 + (1).

1 $$\:\frac{\left[RSsw\right]sensor-\left[RSs\right]standard\:method}{\left[RSs\right]standard\:method}\:)*100$$

where *[RSs]standard method* represents the concentration of RSs obtained using either a standard titration or the value available on the label of the sample.

### Titration of reducing sugar

To compare the results of the developed sensor, a standard Fehling’s titration method [59] was performed to quantify the RSs in real samples. Two Fehling’s solutions were prepared as follows: 70 g/L of CuSO_4_.5H_2_O (Fehling A) and 350 g/L of KNaC_4_H_4_O_6_.4H_2_O combined with 100 g/L of NaOH (Fehling B). For each titration, 5 mL of Fehling A was mixed with 5 mL of Fehling B and 40 mL of deionized water to produce Fehling’s reagent. This reagent, which has a characteristic blue color, was heated to its boiling point. Once boiling, a solution containing reducing sugar was added drop by drop until the color changed to red. Next, two drops of a 1% methylene blue solution were added, causing the entire solution to turn blue again, and it was boiled for a further minute. Following this, another aliquot of the solution containing the reducing sugar was added once more until the blue color completely disappeared (endpoint). The concentration of RSs was calculated using the following equation [60]:

2 $$\mathrm{RSs}\left(\mathrm g/\mathrm L\right)=51.5\ast\mathrm D/\mathrm A$$

where D is the dilution factor (if the case of a diluted sample) and A represents the volume in mL of the solution added until the endpoint was reached. Titrations were performed five times for each sample. According to the procedure, if the volume of the added solution (A) was less than 5 mL, the experiment was repeated with a diluted solution to ensure that more than 5 mL were added to ensure accuracy.

## Results and discussion

**Fig. 1:**
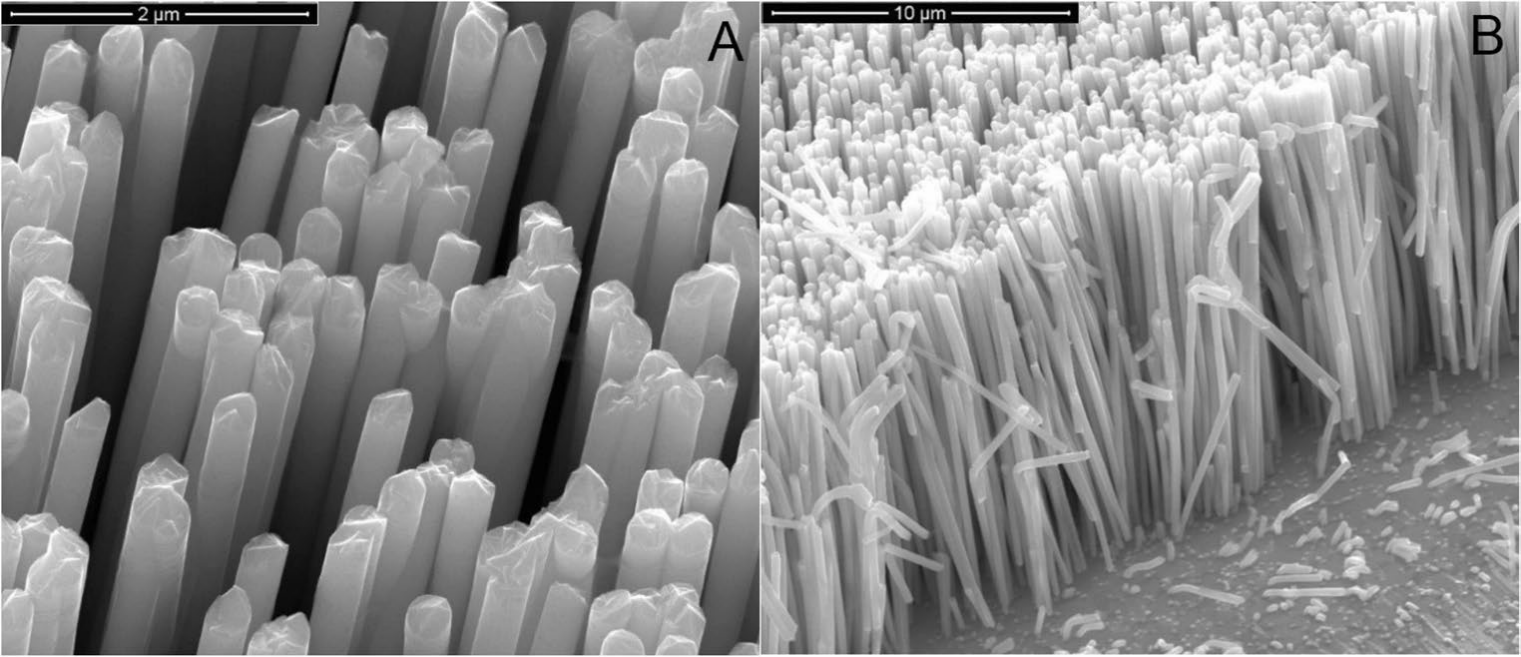
SEM images of NiFe NWs: (**A**) Tilted top view (100000 X); (**B**) Cross-section view (20000 x)

Electrochemical deposition led to a nanostructured electrode consisting of a regular array of vertically standing NWs (see Fig. 1A-B) that covered the entire surface of the Ni current collector (Fig. S3 A). The NWs morphology ensured a very high surface area and thus very high electrocatalytic activity. NWs had a cylindrical shape (mean diameter ~ 246.5 nm ± 16.5, Figure S3B) with smooth and regular wall surfaces. They also presented the typical interconnections due to template morphology. Figure 1-B shows a cross-section of an electrode array with an average length of 11.4 μm ± 0.55 (Figure S3C). From these images, an extremely high surface area NiFe nanostructured electrode was confirmed. EDS analysis (Fig. S3 D) was performed to evaluate the composition of NWs, a Fe content of ~ 79% and a Ni content of 21% was observed, in agreement with our previous results [55]. These percentages differed to the composition of the plating bath (Ni 67%, Fe 33%). NWs were richer in Fe due to the anomalous co-deposition of Fe alloy as reported in [61]. By XRD (Fig. S3 E) the deposition exhibited a face-centered cubic (FCC) Fe-Ni alloy crystal structure (card no. 47–1405), preferentially oriented along the (200) plane.

**Fig. 2:**
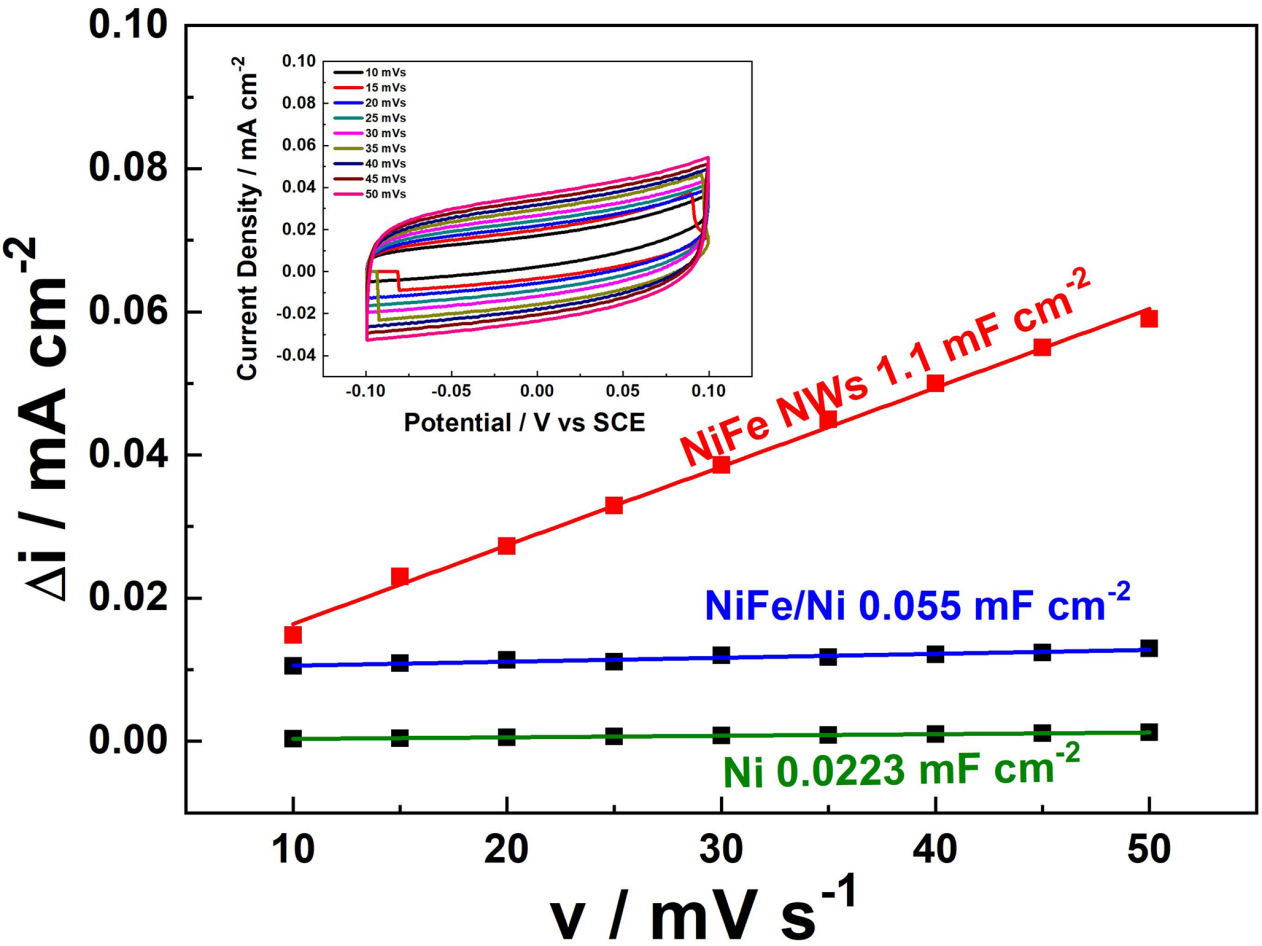
Specific capacitance of the NiFe on Ni foil, and NiFe NWs electrodes evaluated by the double layer capacitance method. Inset: CV at different scan rates of NiFe NWs

The double-layer capacitance (CdL) of the NWs electrode was assessed using cyclic voltammetry (CV) tests conducted at varying scan rates (v). These tests were performed using a 0.1 M NaOH solution within a non-Faradaic potential window (from − 0.1 to 0.1 V vs. SCE). To emphasize the high surface area of the NWs, comparative tests were also carried out using a planar nickel foil and a nickel foil modified by electrodeposition of a thin NiFe alloy film. The NiFe films were deposited under identical conditions to those used for the NWs, ensuring consistent composition and crystalline structure (Fig. S3 D-E). The NiFe film exhibited clusters of nanoparticles that were uniformly distributed across the Ni foil surface (Fig. S4 A-B). For all electrodes analyzed, the plots of the difference between anodic and cathodic current density (∆i = (i_A_-i_C_), measured at 0 V vs. SCE) exhibited a linear increase with scan rate, as shown in Fig. 2. Since the slope of the ∆i plot directly correlates with specific capacitance, the results revealed that the CdL of the NiFe NWs electrode is nearly 50 times greater than that of the planar Ni foil electrode and 20 times greater than that of the NiFe alloy modified Ni foil. Given that CdL is intrinsically linked to the electrochemically active surface area, these findings underscore the conclusion that NiFe NWs possess a high active surface area and exhibit remarkable electroactivity compared to the control samples.

The reducing sugar reaction on a NiFe NWs electrode was investigated by CV performed at different scan rates by using a solution of 0.1 M NaOH and 5 mM of glucose as a model sugar. Before these tests, a NiFe electrode was first activated by undertaking 10 CV cycles in 0.1 M of NaOH or until overlapped CV curves occurred.

**Fig. 3:**
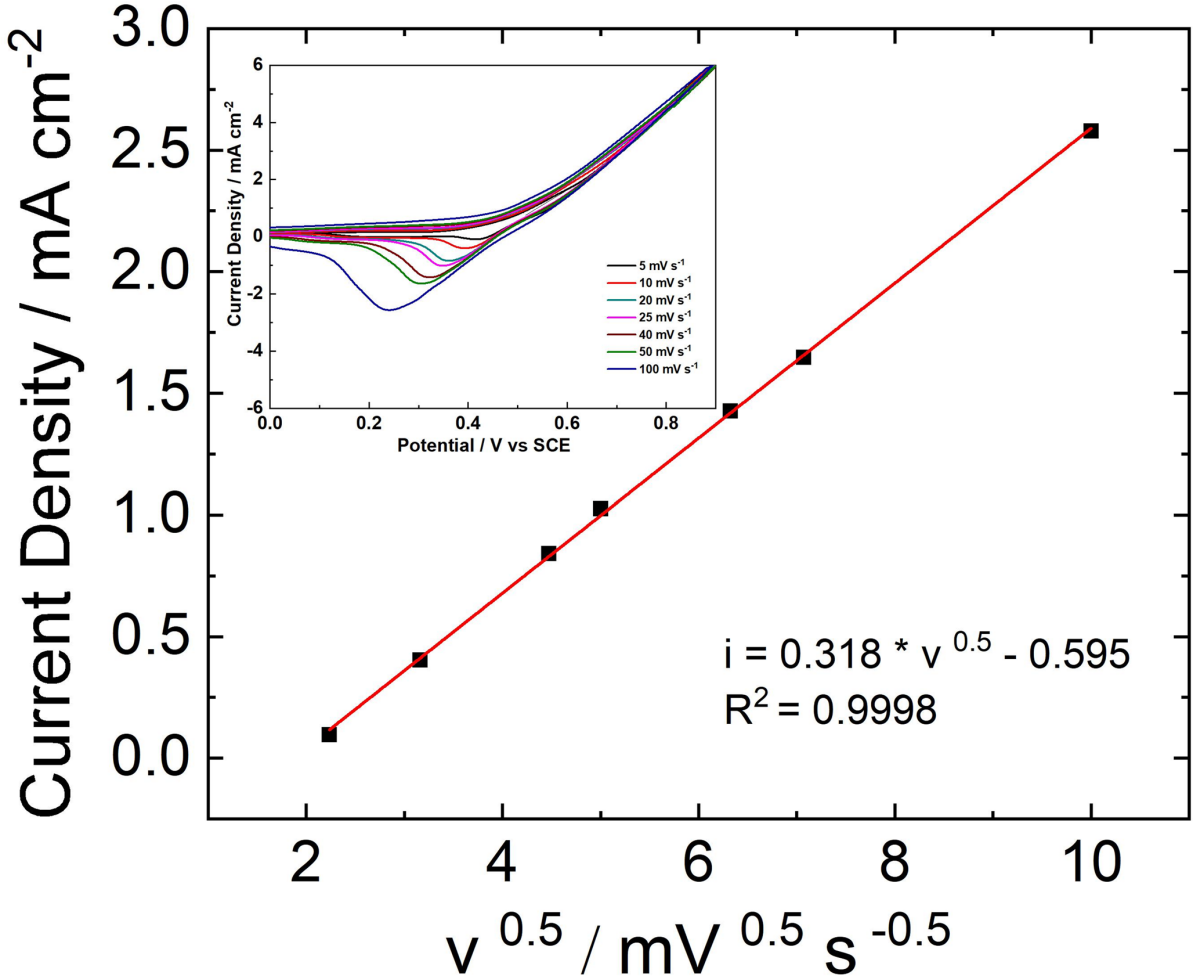
Effect of scan rate on CV response of 5 mM glucose in 0.1 M NaOH

As can be observed in Fig. 3, the increase of peak current density is linear with the square root of the scan rate. This suggests a diffusion-controlled process in agreement with the literature data [45]. The inset of Fig. 3 shows the effect of scan rate from 5 to 500 mV s^–1^ on the CVs in the presence of 5 mM glucose. With the increase in scan rate the peak intensity increases and shifts toward lower potential values, arising from the increase of overpotential towards glucose electro-oxidation [45]. The same experiment was carried out using other reducing sugars with very similar results, suggesting a similar diffusion-limited behavior.

**Fig. 4:**
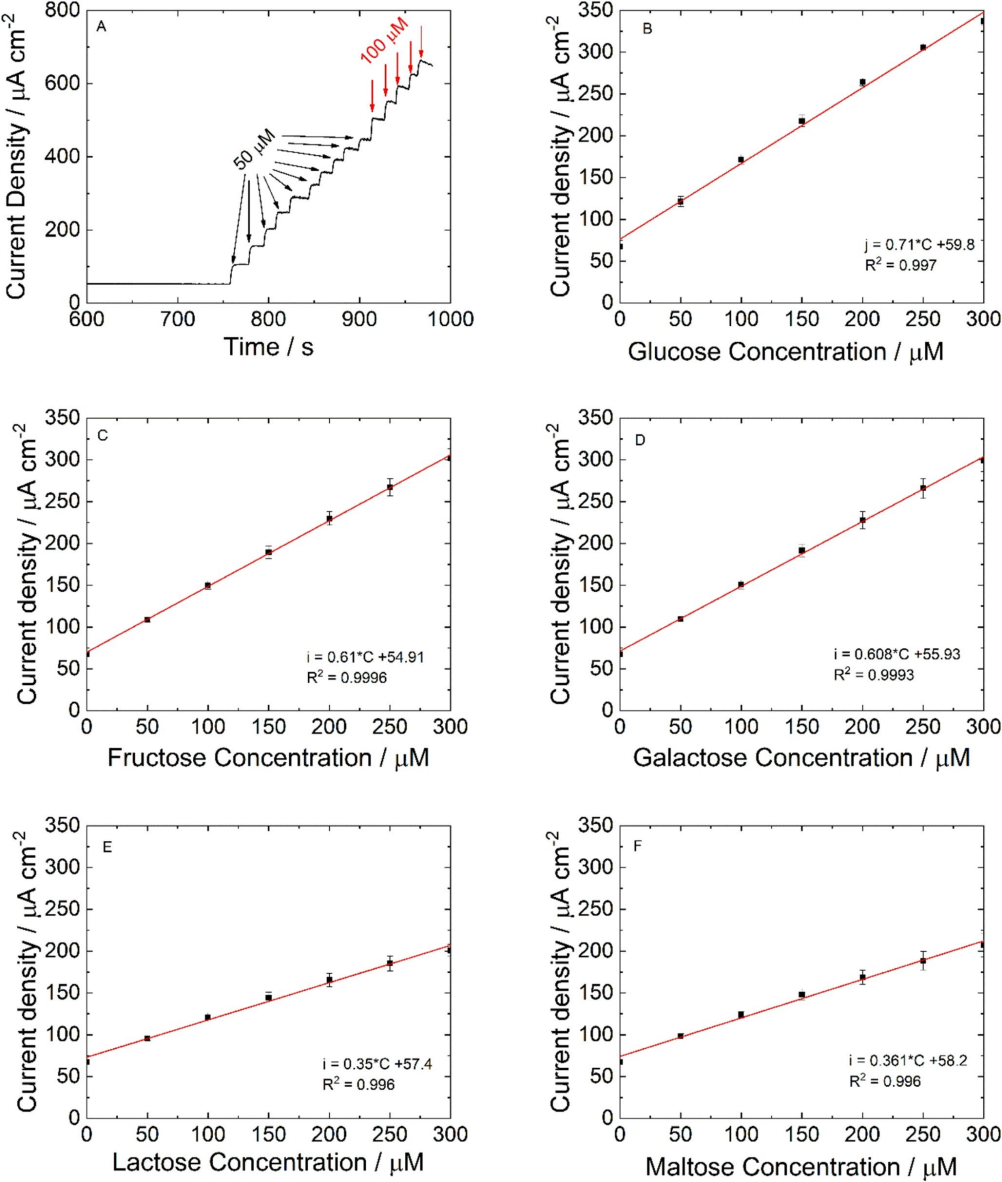
(**A**) Chronoamperometric response for glucose detection at 0.5 V vs. SCE in 0.1 NaOH stirred solution. Calibration lines: (**B**) Glucose, (**C**) Fructose, (**D**) Galactose, (**E**) Lactose, (**F**) Maltose. Error bars represent the SD of five measurements

The irreversible oxidation of glucose on NiFe electrodes follows the model reported in [45]. In alkaline solutions, the first stage is metal oxidation to metal hydroxides (M^II^(OH)_2_) followed by further oxidation to metal oxyhydroxide or oxide (M^III^OOH or M^IV^O_2_). Upon exposure to glucose, the M^III^ or M^IV^ sites act as a catalytic site for the oxidation of glucose to gluconolactone (the reactions in the Supplementary Materials). The result is the generation of an anodic current proportional to the concentration of reducing sugar present in the electrolyte. The Fe component facilitates electron transfer and increases the density of active sites, enhancing both catalytic activity and sensitivity. A similar oxidation mechanism occurs using other reducing sugars, but with the formation of different end products. The performance of reducing sugar sensors was evaluated by chronoamperometry (CA) at + 0.5 V vs. SCE. This potential was selected from the CV tests that were performed at a very low scan rate (v = 0.5 mV s^− 1^, Fig. S5A). Once the current reached a steady state, increasing amounts of reducing sugar were added by pipetting and mixing them into the cell through the pump and microfluidic channels. Figure 4A shows a CA test undertaken with increasing glucose concentration. In the blank solution, a baseline current (~ 55 µA cm⁻²) was measured, attributed to NiFe oxidation. The current density increased with increasing glucose concentrations, suggesting good electrocatalytic performance of the electrodes. The average response time was 6 s. Similar experiments were carried out for fructose, galactose, lactose, and maltose. All measurements were performed five times, and the main values of current density were reported with standard deviation. The concentration range from 0.05 to 1 mM was investigated. In this range, three different linear ranges were observed, each with different sensitivity values that decrease with increasing sugar concentration, as expected for non-enzymatic sensors [62] (Fig. S5B). For all reducing sugars, the first linear range extended from 0.05 to 0.3 mM. In Figs. 4B-F the relative calibration lines are reported. The limit of detection (LOD) and limit of quantification (LOQ) were calculated using the following equations:

3 $$\:LOD=3.3\:SD\:{S}^{-1}$$

4 $$\:LOQ=10\:SD\:{S}^{-1}$$

where SD is the standard deviation of the blank and S is the slope of the linear calibration curve. The calculated values are reported in Table 1, where the mean features of the sensor are summarized. To evaluate the repeatability and reproducibility of the sensor, the calibration was performed five times with the same electrode and five times with a different electrode (Table 1).

**Table 1:** Sensor performance obtained for reducing sugars using NiFe NWs-based electrode

RS	Linear Range	Sensitivity µA µM^− 1^ cm^− 2^	Repeatability %	Reproducibility %	LOD µM	LOQ µM
Glucose	0.05–0.3 mM	0.71	1.32	6.2	2.31	7.74
Fructose	0.05–0.3 mM	0.61	5.03	7.3	2.7	8.15
Galactose	0.05–0.3 mM	0.608	2.55	10.3	2.72	8.2
Lactose	0.05–0.3 mM	0.350	4.97	5.98	4.71	14.28
Maltose	0.05–0.3 mM	0.361	4.91	6.3	4.57	13.85
Mean of monosaccharides	0.05–0.3 mM	0.642 ± 0.058	2.96 ± 1.88	7.91 ± 2.1	2.57 ± 0.22	8.03 ± 0.25
Mean of disaccharides	0.05–0.3 mM	0.355 ± 0.0078	4.94 ± 0.04	6.14 ± 0.22	4.62 ± 0.12	14 ± 0.3

Results showed that for glucose, fructose, and galactose, (monosaccharide sugars), the sensitivities were very similar, suggesting that reducing sugars react similarly at the surface of the NiFe NWs. Interestingly, lactose and maltose (disaccharide sugars) have similar behavior but a lower sensitivity when compared to monosaccharide sugars. This is attributable to their lower diffusion coefficients and larger molecular size, which restricts their ability to access active sites. The reduced diffusion coefficient of these reducing sugars is a consequence of their higher molecular weight, as diffusion coefficients generally decrease with increasing molecular weight; although the relationship varies depending on the type of molecule and solvent [63]. Additionally, the large molecular size of disaccharides introduces steric hindrance, further limiting their ability to reach active sites. This, in turn, affects detection, as the oxidation reaction of a reducing sugar occurs at specific functional groups (aldehyde or ketone) of the molecule [64]. These aspects make the reaction of disaccharide sugars more difficult than that of monosaccharide sugars, justifying the lower sensitivity. Therefore, we can conclude that the proposed sensor can quantify different RS, with different sensitivities for monosaccharides (higher) and disaccharides (lower). In any case, for all investigated sugars the LOD values are low enough to allow the use of the NiFe NW-based sensor for both the food industry and healthcare applications. In addition, the results show good reproducibility and repeatability between electrodes *n* = 5.

**Fig. 5:**
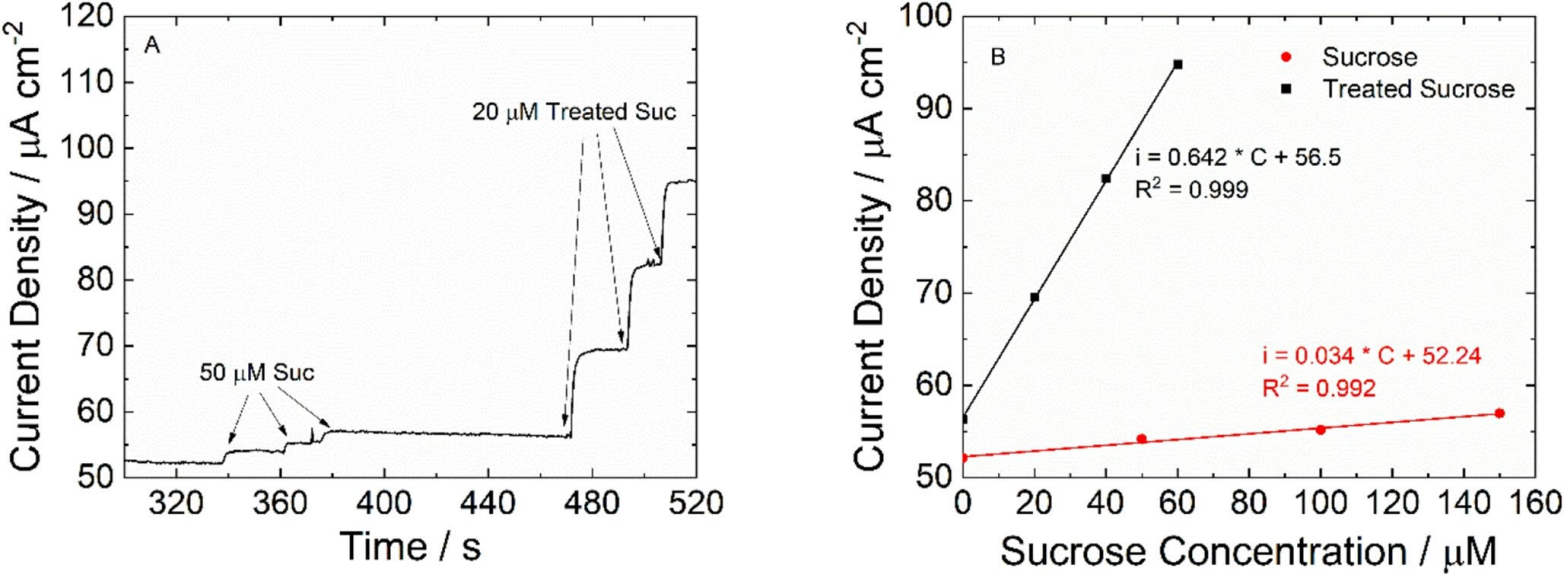
(**A**) Chronoamperometric response for treated and not treated sucrose and (**B**) corresponding calibration lines. Error bars represent the SD of five measurements

Before analyzing the real samples, an interference study was carried out using different species, including sucrose, which is a non-reducing sugar. The injection of different amounts of sucrose into the solution (Fig. S6A) leads to a very small increase in the current density, suggesting that NiFe has a low activity for non-RSs. A sensitivity for sucrose of 0.034 µA µM^− 1^ cm^− 2^ was calculated (Fig. S6B), which is more than 10 times lower than the mean of disaccharide reducing sugars and 20 times lower than the mean of monosaccharides. This result suggests that the NiFe-based sensor is selective towards reducing sugars. The possibility of detecting even non-reducing sugars was also studied. For this aim the sucrose solution was pretreated by adding 1 M HCl and heated to 60 °C for 1 h. This procedure allowed the splitting of sucrose into glucose and fructose units, making their quantification as RSs. The results reported in Fig. 5A confirm the validity of this procedure. The response of the sensor for a non-treated 50 µM sucrose solution is very low, while the addition of 20 µM of the treated solution led to a remarkable increase in current density that can be attributed to the splitting of glucose and fructose. Moreover, as can be observed in Fig. 5B, the slope of the calibration line obtained for the treated sucrose solution is almost 20 times higher than the untreated sucrose and with a value that is very close to those obtained for glucose and fructose (Figs. 4B and C).

**Fig. 6:**
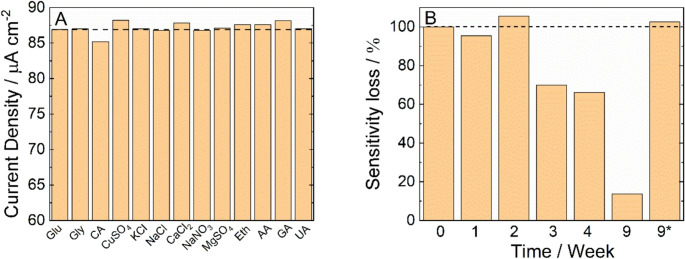
(**A**) Selectivity test towards different interferant species in the presence of 50 µM RS (**B**) NiFe stability evaluated for glucose sensing. *after the re-oxidation process

Reducing sugar selectivity was also assessed in the presence of many species (not sugars) typically present in different foodstuffs (KCl, NaCl, citric acid, copper sulfate, uric acid, calcium chloride, magnesium sulfate, sodium nitrate, glycerin, and ethanol). These potential interferents were injected sequentially into a solution containing 50 µM of glucose and the response was recorded. To explore the worst-case scenario (very high concentrations of potential interferents were employed), the concentration of each interferent was selected starting from its maximum concentration value in the real sample multiplied by a safety factor (**≥** 20). The most notable variations in sensor response were observed in the presence of copper ions, citric acid, as illustrated in Fig. 6. However, it is important to emphasize that the concentrations used for citric acid and copper ions were intentionally exaggerated, with safety factors of 250 and 350 (respectively) well above real and relevant levels. Despite these extreme conditions, the sensor displayed only minimal current changes, indicating a very low level of interference from these compounds. For all other interfering substances tested, even at extremely high safety factors exceeding 1000x, no measurable interference with glucose detection was observed. These results provide strong evidence that the NiFe NWs-based sensor exhibits excellent selectivity for reducing sugars, even in the presence of potentially interfering species at high concentrations.

Selectivity was also assessed for some natural antioxidant species in different foodstuffs. In particular, gallic and ascorbic acid have been tested. As can be observed in Fig. 6A, a minor interference of the sensor was observed for these species. However, the observed increase in current was marginal and can be considered negligible (Figure S7).

In Fig. 6B, the stability of the NiFe electrode was shown. Sensor sensitivity was evaluated by calibrating the electrode every week after simple storage in air at room temperature. It can be observed that during the first two weeks, the sensor remains perfectly stable, maintaining its initial sensitivity. After this period, the sensitivity gradually decreases, reaching very low values after nine weeks. However, this is not a critical issue, as the electrode can be easily reactivated to restore the catalytic sites for glucose oxidation [65] and thus its initial sensitivity. The reactivation procedure is straightforward, consisting of a polarization at 0.8 V vs. SCE for 45 min at room temperature, and it can be performed directly in the measurement cell prior to the test using the same blank solution employed for sample dilution. In conclusion, the electrode is stable and can be reused multiple times following this simple reactivation step.

**Fig. 7:**
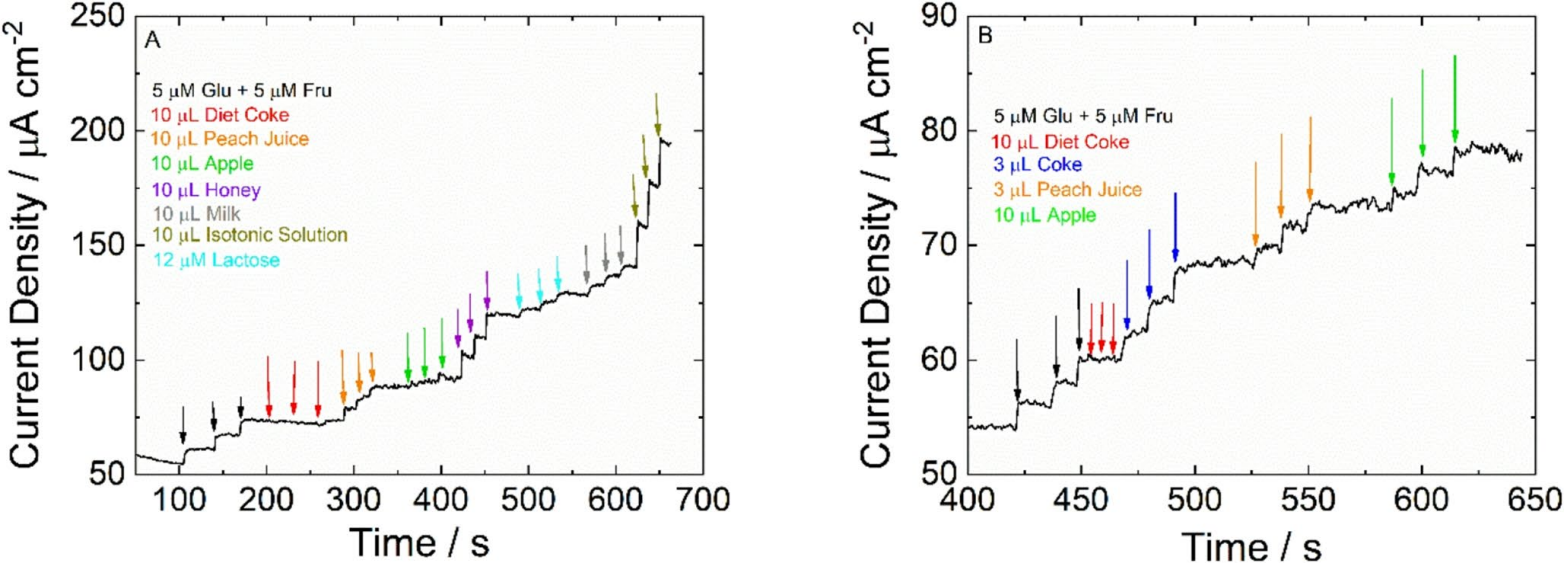
Real sample analysis using the NiFe NWs-based sensor. (**A**) Test for only RS of Diet Coke, Coke, Peach Juice, Honey, extracted Apple, isotonic solution, and milk; (**B**) Test for total sugar content after sample treatment (acidification and heating) of Diet Coke, Coke, Peach juice, and Extracted Apple

To assess the efficacy of NiFe-NWs-based sensors for the detection of reducing sugar concentrations in real sample matrices, different foods (diet coke, regular coke, peach juice, honey, isotonic solution, extracted apple, and milk) were tested and the results are presented in Fig. 7. For electroanalysis, food samples were diluted in 0.1 M NaOH to reach a similar pH value to that used for the calibration line. Dilution is also necessary due to the high concentration of sugars in real samples. The reducing sugar concentration of the food samples was calculated using the mean of the calibration of monosaccharides. In the case of milk, given that the main reducing sugar is lactose, the sugar concentration was calculated using the calibration line obtained for lactose (Fig. 4E). In particular, 0.1 M NaOH was inserted into the electrochemical cell while pumping. After signal stabilization, different aliquots of food sample (previously diluted) were pipetted into the solution and made it mix through the pumping and the microfluidic channels. The sensor results were compared with those obtained with either standard titration or written on the sample label (the labels were reported in the Supplementary Materials) and exhibited excellent correlation strongly suggesting the sensors were not affected by the sample matrix. Results are summarized in Table 2 and recovery was calculated according to Eq. 2 (NT denotes not tested). The very high values of recovery suggest good behavior of the sensor. In addition, comparable results with standard methods were obtained.

**Table 2:** Food sample analysis of RS and non-RS using the NiFe NWs-based sensor and comparison with standard method

Food	RS with sensor	SM	RS with SM	REC %	T-RSs with sensor	SM	T-RSs with SM	REC %
Peach Juice	44.72 ± 0.4 g/L	TT	46.88 ± 2.1 g/L	95.4	137 ± 16 g/L	LB Fig. S10	140 g/L	97.85
Honey	0.756 ± 0.03 g_RS_/g_honey_	TT	0.79 ± 0.004 g_RS_/g_honey_	96.1	NT	NT	NT	NT
Coke	NT	NT	NT	NT	123 ± 17 g/L	LB Fig. S8	120 g/L	102.5
Diet Coke	0 g/L	TT	0	100	0	LB Fig. S9	0	100
Milk	52.6 ± 2.3 g/L	LB Fig. S11	50 g/L	105.2	NT	NT	NT	NT
Isotonic Solution	48.8 ± 3.1 g/L	LB Fig. S12	50 g/L	97.5	NT	NT	NT	NT
Apple	75.1 ± 0.87 mg_RS_/g_apple_	Ref [66]	79.82 ± 15.9 mg_RS_/g_apple_	94.1	122 ± 0.72 mg_RS_/g_apple_	Ref [66]	127.3 ± 29.8 mg_RS_/g_apple_	96.1

*SM*: standard method, *TT*: Titration, *REC*: Recovery, *T-RSs*: Total Sugar, *LB*: Label, *NT*: Not tested, *NC*: not calculated

The results presented in Table 2 highlight that NiFe NWs-based sensors can be used for real-time analysis, making them ideal for on-site quality control in the food industry. Sample analysis can be conducted directly at the production and/or handling site of the food, eliminating the need for specialized laboratories. By utilizing the proposed sensor, it is sufficient to take a very small quantity of the sample and transfer it directly into the analysis cell, which already contains the basic solution for dilution.

The performance of the NiFe NWs-based sensor was compared to that of other non-enzymatic electrochemical sensors (Table 3) proposed in the literature for sugar quantification. Although sensors with lower LOD can be found, the NiFe NWs-based sensor developed here can quantify sugars in a wide range of concentrations and with a very high sensitivity. This aspect is much more important for sugar detection; indeed, sugar concentration is quite high (mM range) in almost every sample. This means that a LOD in the µM range is low enough to be used in every application and thus an improvement in this parameter is unnecessary. Contrary, the higher the sensitivity, the higher the accuracy and the precision of the sensor.

**Table 3:** Comparison with other sensors for RSs electrochemical detection based on nickel and nickel alloys

Sensor	T	RS	L.*R*.	S.	LOD	I	Re. Sa.	Ref.
Ni-NDA/CNTs/GCE	CH	FRU	10^− 3^−0.12	10^− 4^	0.19	AA, UA	AJ, OJ, LJ	[67]
NiCr	CH	GLU, RIB, MAL, SUC	0.01–0.55	1.37	3	NS	NS	[68]
CoNi	CH	GLU	1–7.6.6	0.97	0.6	MAN, FRU, UA, AA, DA, ST, 4AC	SE	[42]
CuNi	CH	GLU	0–10^3^	0.24	NS	UA, AA	NS	[69]
NiFe/GO/GCE	CH	GLU	0.05–5.05	0.17	9	UA, AA, DA	SE	[70]
NiFe (NPs) - PANi	CH	GLU	0.02–1.02	1.05	0.5	AA, SAC, KCl	NS	[45]
CS-rGO - NiNPs	CH	GLU	0.2–9.2	0.318	4.1	UA, AA, DA	UR	[71]
GC/MWCNT/NiO	CV	GLU	0.2–12	0.436	160	N.S.	SE	[72]
NiNFs-SPEs	CH	GLU, FRU	0.025–1.025	0.21	8	CA, LA, AA, ETh, GLY	OJ, HN	[73]
NiCo NPs	CH	GLU	0.001–1.001	0.853	0.175	Na^+^,Cl^−^,Mg^2+^,Ca^2+^,FRU, AA, SO_4_^2−^,DA, H_2_O_2_,UA	Sa	[74]
CoNi_2_S_4_@NCF	CH	GLU	0.5–12.5	0.0067	NS	AA, DA, KCl, NaCl, FRU, LA, UA, Urea,	SE	[75]
GO-modified Ni foam	CH	TR		0.00166	37.1	Na_2_CO_3_	NS	[76]
Ni(OH)_2_-SPGE	CV	GLU	0.01–0.2		1.8	D-GLU, FRU, AA, MAL	AJ, SJ, OJ, CK, sprite	[77]
NiFe NWs	CH	GLU, FRU, LAT, MAL, GAL, SUC,	0.05–0.3	0.71	2.3	AA, CA, CuSO_4_, KCl, NaCl, CaCl_2_, ETh, MgSO_4_, Gly, NaNO3	CK, CK0, AJ, PJ, IS, MK, HN	This work

*4AC*: 4-acetominophenol, *AA*: ascorbic acid, *AJ*: Apple juice, *CA*: citric acid, *CH*: chronoamperometry, *CNT*s: carbon nanotubes *CS*: chitosan, *CK*: coke, *CK0*: diet coke *CV*: ciclic voltammetry, *DA*: dopamine, *ETh*: ethanol, *FRU*: fructose, *GAL*: Galactose, *GCE*: Glassy carbon electrode. *GLY*: Glycerol, *GLU*: glucose, *GO*: graphene oxide, *HN*: honey, *I*: interfering species, IS: isothonic solution, *LAT*: lactose, *LOD*: limit of detection [µM], *LR*: linear range [mM], *LJ*: lemon juice, *MAL*: maltose, *MAN*: Mannose, *MK*: Milk, *MWCNT*: multi walled carbon nanotubes, *NF*s: nanoflowers, *NS*: not studied, *Ni-NDA*: Ni-naphthaline-1,4-dicarboxylic acid, *NP*s: nanoparticles, *NW*s: nanowires, OJ: orange juice, *PANI*: polyaniline, PJ: peach juice, *rGO*: reduced graphene oxide, Re. *Sa*.: Real samples, RIB: ribose, *RS*: Reducing sugar, *S*: sensitivity [µA µM^− 1^cm^− 2^], *Sa*: Saliva, *SE*: serum, *SPGE*: screen-printed gold electrode, *SJ*: sourcherry juice, *ST*: Serotonine, *SUC*: sucrose, *T*: technique, *TR*: trehalose, *UA*: uric acid, *UR*: urine

## Conclusions

NiFe NWs electrodes were fabricated using a template electrosynthesis method and demonstrated selective and sensitive detection of different reducing sugars. The physical chemical characterization of sensors showed a regular array of NiFe NWs with an iron content of ~ 79%. This morphology ensured a high electrochemically active surface area, confirmed through evaluation of double-layer capacitance. Cyclic voltammograms at varying scan rates exhibited a diffusion-controlled process. For each reducing sugar a calibration line was constructed using chronoamperometry at 0.5 V versus SCE. Three different linear ranges were found. For all investigated sugars, the first linear range extended from 0.05 to 0.3 mM with LOD values in the µM (2.57 and 4.62 µM for mono and disaccharides, respectively) scale with R^2^ > 0.99. For monosaccharide sugars, a sensitivity approximately doubles that of disaccharide sugars was found. Sensors were not suitable for non-reducing sugars, which could, however, be quantified after a simple pre-treatment of the sample by acidification and heating. The effects of interfering species commonly found in foods and tested at very high concentrations were investigated, demonstrating excellent sensor selectivity. The sensor also has good reproducibility and repeatability. Finally, a validation of the sensor with real samples was performed, resulting in very high recovery values (recovery ranging from 95 to 105%). In addition, a very good agreement between the sensor results and those from conventional techniques was obtained. All the results demonstrated that the NiFe nanowire-based sensors are very promising for the application of both reducing and non-reducing sugar detection. Despite the promising results, several challenges remain in the development of NiFe nanowire-based electrochemical sensors for total sugar detection in food matrices. Future studies should focus on optimizing the Ni/Fe composition to enhance the catalytic activity and selectivity toward reducing sugars. Surface modification (by coating deposition or functionalization) could be explored to improve electrode stability and minimize fouling in complex food matrices. Moreover, the integration of NiFe nanowire-based sensors into portable platforms would enable rapid and in situ determination of total sugars. Addressing these issues will be essential to translate the laboratory-scale NiFe electrodes into practical and on-site applications.

## Supplementary Information

Below is the link to the electronic supplementary material. 

Supplementary Material 1 (DOCX. 3.44 MB)

## Data Availability

No datasets were generated or analysed during the current study.
