# Supplementary material for: Sensitive electrochemical detection of total sugars in food using NiFe alloy nanowires
Source: Mikrochim Acta. 2025 Nov 17;192(12):819. doi: 10.1007/s00604-025-07663-3 (PMC12628486; doi:10.1007/s00604-025-07663-3)
Supplement: Supplementary file 1 — Supplementary Material 1 (DOCX. 3.44 MB) [file 604_2025_7663_MOESM1_ESM.docx]

**Sensitive electrochemical detection of total sugars in food using NiFe alloy nanowires**

**B. Patella^a*#^, N. Moukri^a*^, F. Mazzara^a^, S. Carbone^a^, R. L. Oliveri^a^, A. Aiello^a^, M. Russo^b^, C. Torino^c^, A. Vilasi^c^, V.B. Juska^d^, A. O'Riordan^d^, R. Inguanta^a^**

^a^ Dipartimento di Ingegneria, Università degli Studi di Palermo, Viale delle Scienze, Palermo, 90128, Italy

^b^ Dipietro Group, Melilli, 96010, Italy

^c^ Institute of Clinical Physiology, National Research Council, Reggio Calabria, 89124, Italy

^d^ Precision Electrochemical Nanosensor Group, Tyndall National Institute, University College Cork, Cork, T12 R5CP, Ireland

^*^ These authors contributed equally to this work.

^#^ Corresponding author: bernardo.patella@unipa.it

**Supplementary Materials**


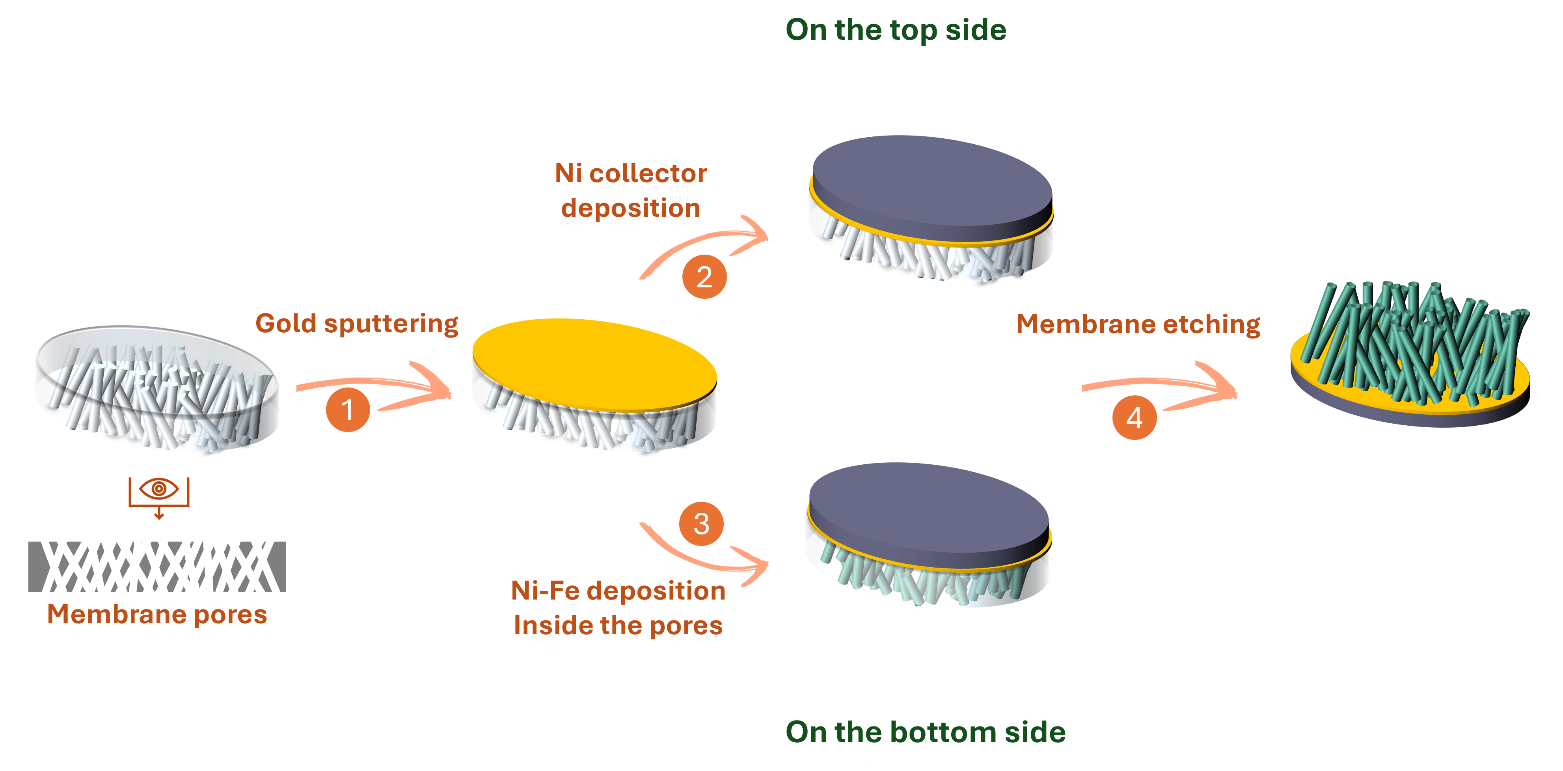


*Figure S1. Scheme of NiFe NWs fabrication process*


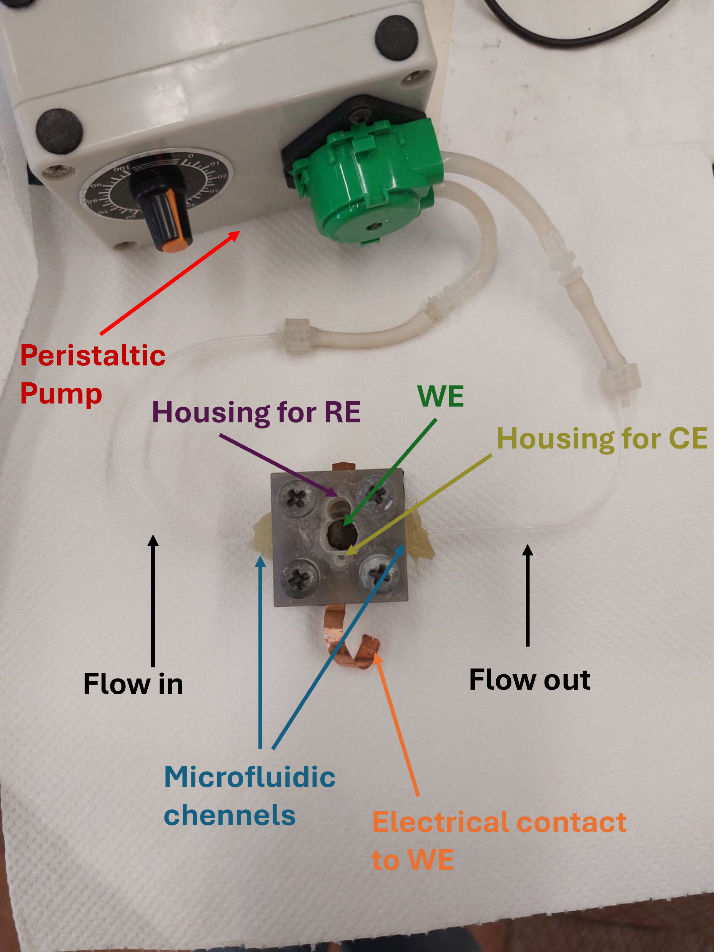


*Figure S2. Optical image of the 3D printed cell with microfluidic channels integrated with WE and a peristaltic pump*


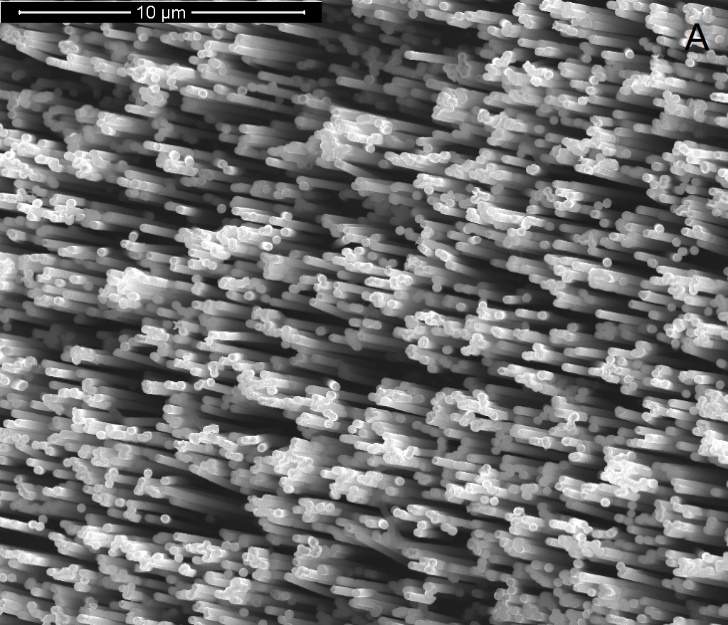

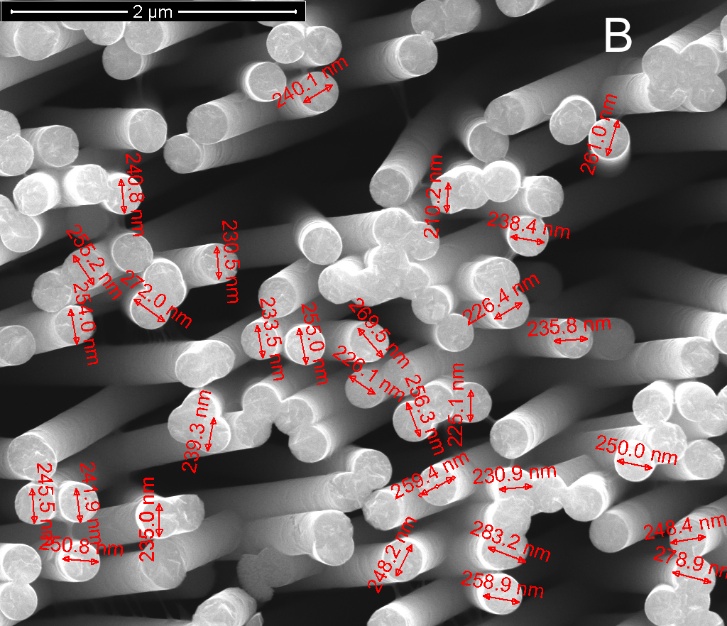

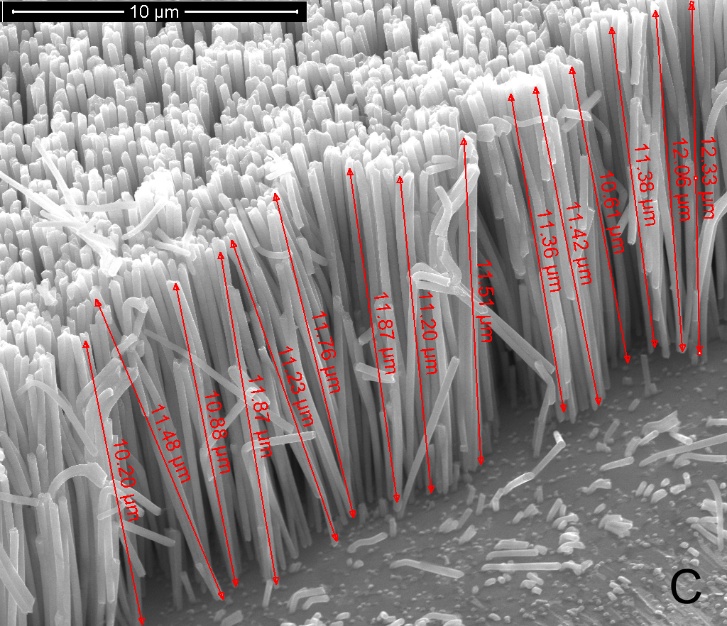






Figure S3. NiFe NWs electrode: (A) SEM images of the top view, (B) SEM image of the top view of NWs and diameter measurements (50000x), (C) SEM image of the cross-sectional view of NWs and length measurements (10000x). (D) EDS spectrum, (E) XRD pattern

.


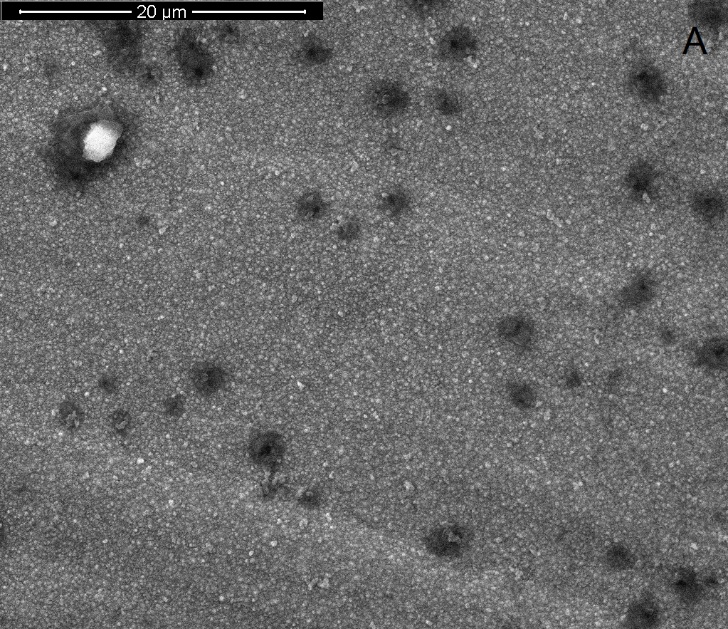

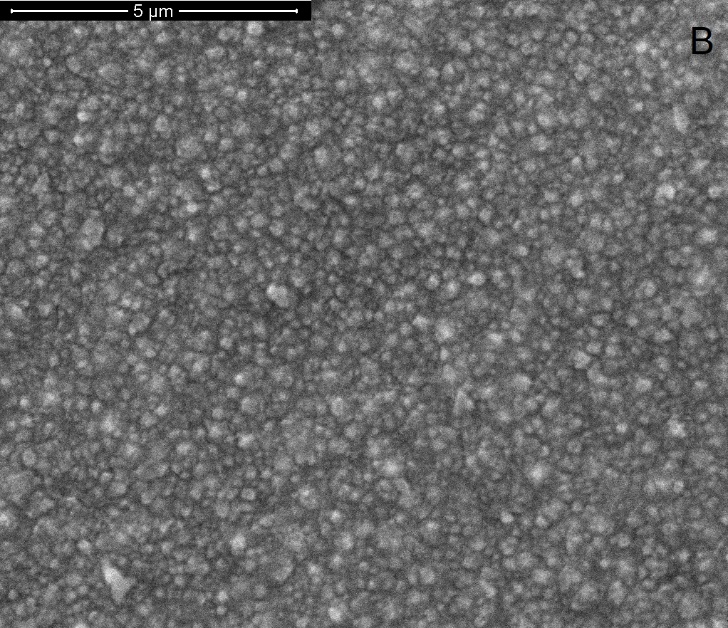


Figure S4. SEM images of NiFe alloy electrodeposited on Ni foil.

**Glucose oxidation mechanism on NiFe electrodes.**

$Ni+{2OH}^{-}\to{Ni(OH)}_{2}{+2e}^{-}$ Reaction 1a

$Fe+{2OH}^{-}\to{Fe(OH)}_{2}{+2e}^{-}$ Reaction 1b

${Ni(OH)}_{2}+{OH}^{-}\to{NiOOH+H}_{2}O{+e}^{-}$ Reaction 2a

$NiOOH+{OH}^{-}\to{NiO_{2}+H}_{2}O{+e}^{-}$ Reaction 2b

${Fe(OH)}_{2}+{OH}^{-}\to{FeOOH+H}_{2}O{+e}^{-}$ Reaction 2c

$FeOOH+{OH}^{-}\to{FeO_{2}+H}_{2}O{+e}^{-}$ Reaction 2d

$NiOOH+glucose\to{Ni(OH)}_{2}+gluconolactone$ Reaction 3a

$NiO_{2}+glucose\to{Ni(OH)}_{2}+gluconolactone$ Reaction 3b

$FeOOH+glucose\to{Fe(OH)}_{2}+gluconolactone$ Reaction 3c

$FeO_{2}+glucose\to{Fe(OH)}_{2}+gluconolactone$ Reaction 3d

According to [1–4], the model involves both M^III^ or M^IV^ catalytic sites where the oxidation of glucose on gluconolactone occurs. A similar oxidation mechanism occurs using other RSs. Particularly, fructose is oxidized to arabinonic acid [5], galactose to formic acid [6], lactose to lactobionate [7] , and maltose to saccharic acids [8]







Figure S5. A) CV recorded at 0.5 mV/sec in the presence of glucose; B) Calibration lines for glucose detection at 0.5V vs SCE in 0.1 NaOH stirred solution. Error bars represent the SD of three measurements.







Figure S6. A) Chronoamperometric curve for sucrose and B) Corresponding calibration line. Error bars represent the SD of three measurements.





*Figure S7 Selectivity test towards different species in the presence of 50 µM RS*

*
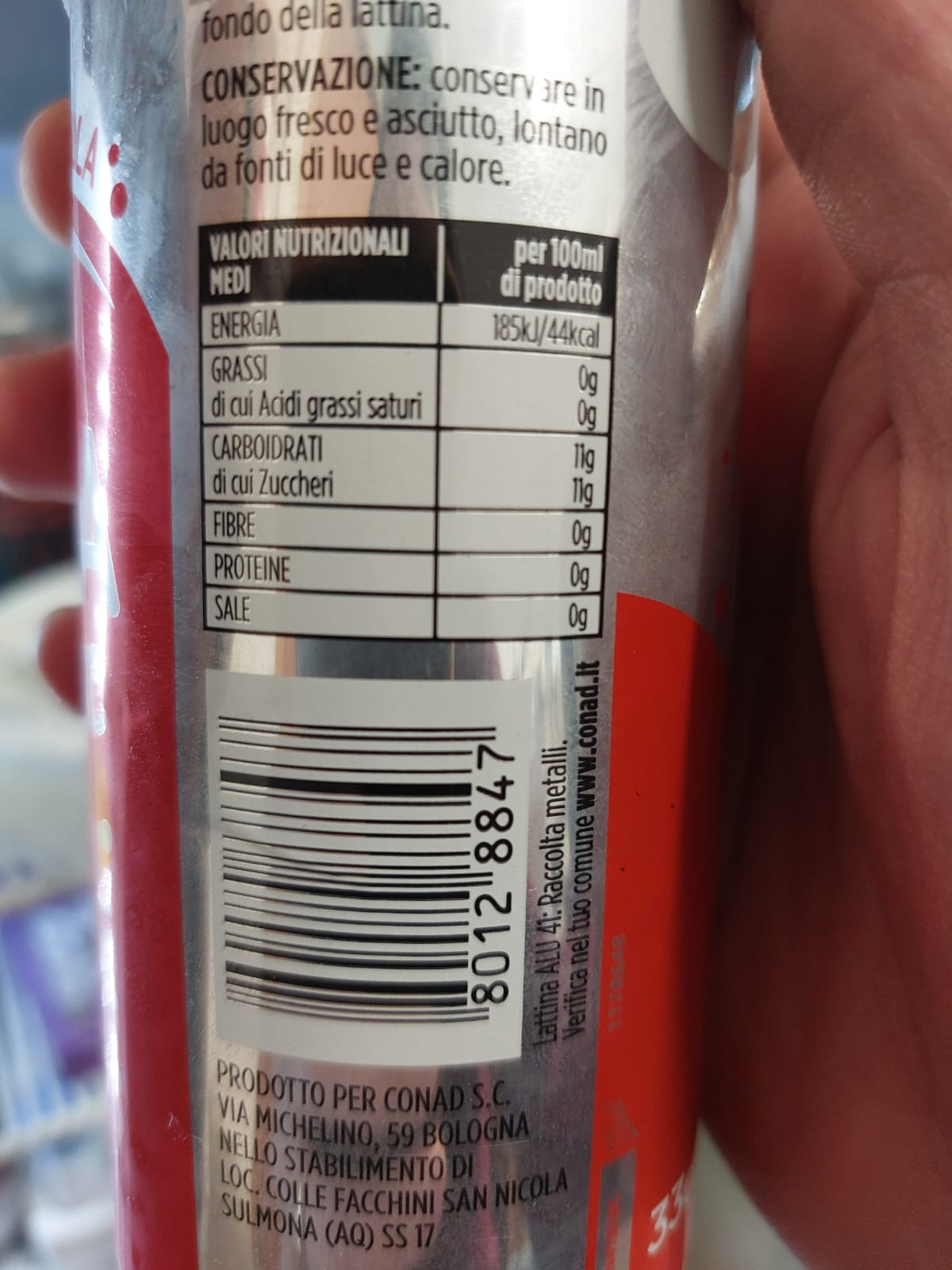
*

*Figure S8. Label of Regular Coke*

*
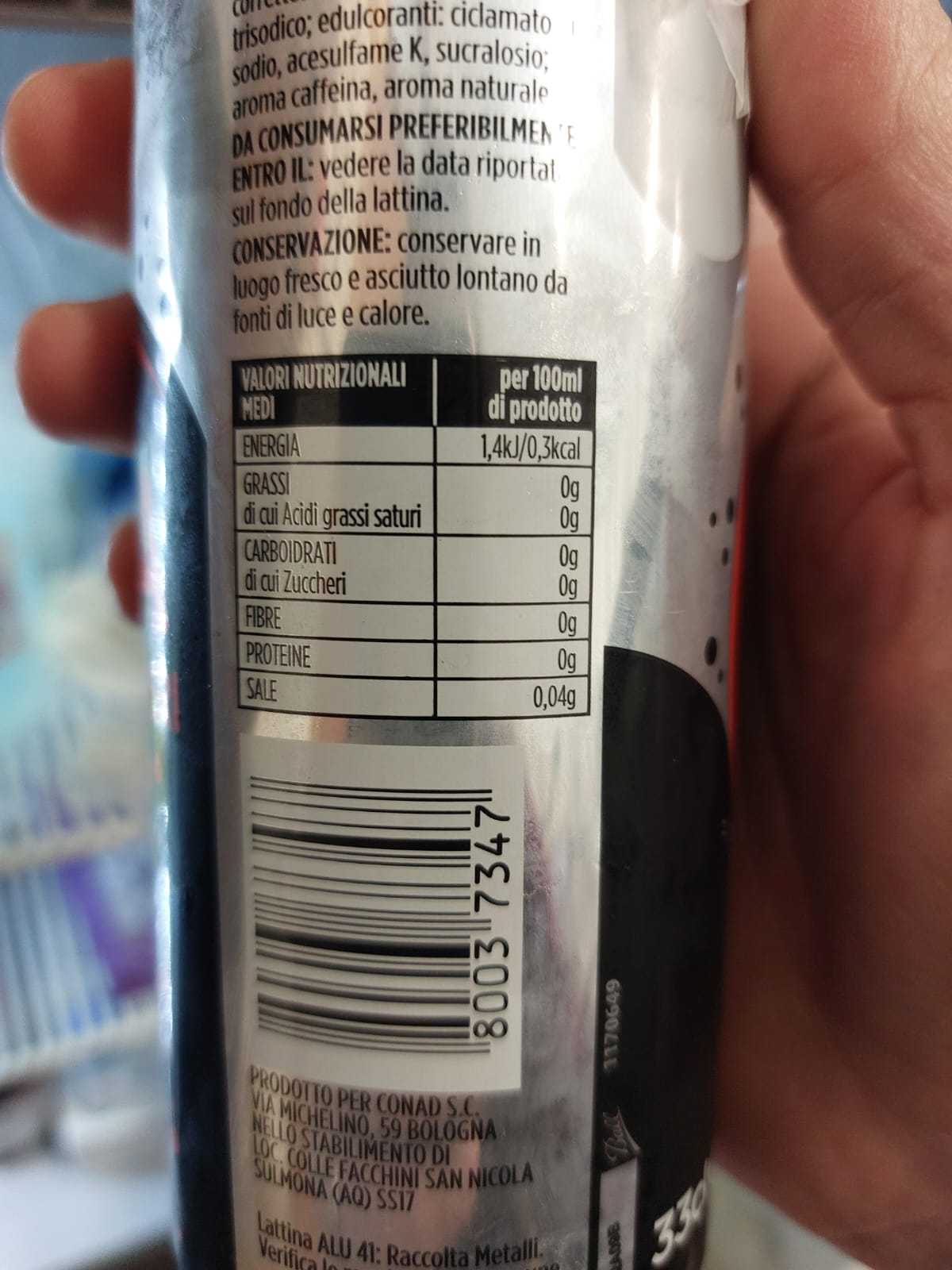
*

*Figure S9. Label of Diet Coke*

*
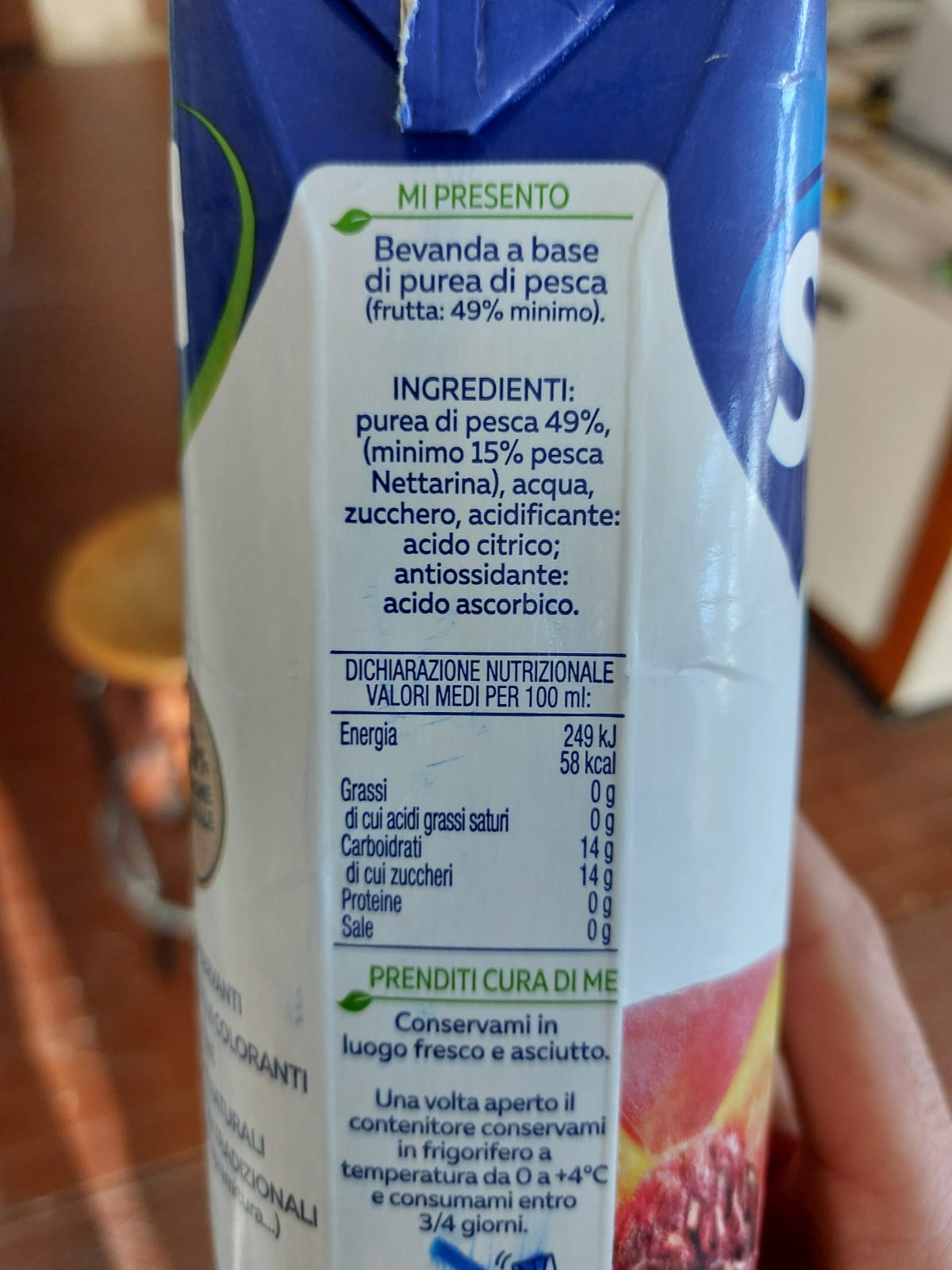
*

*Figure S10. Label of Peach Juice*

*
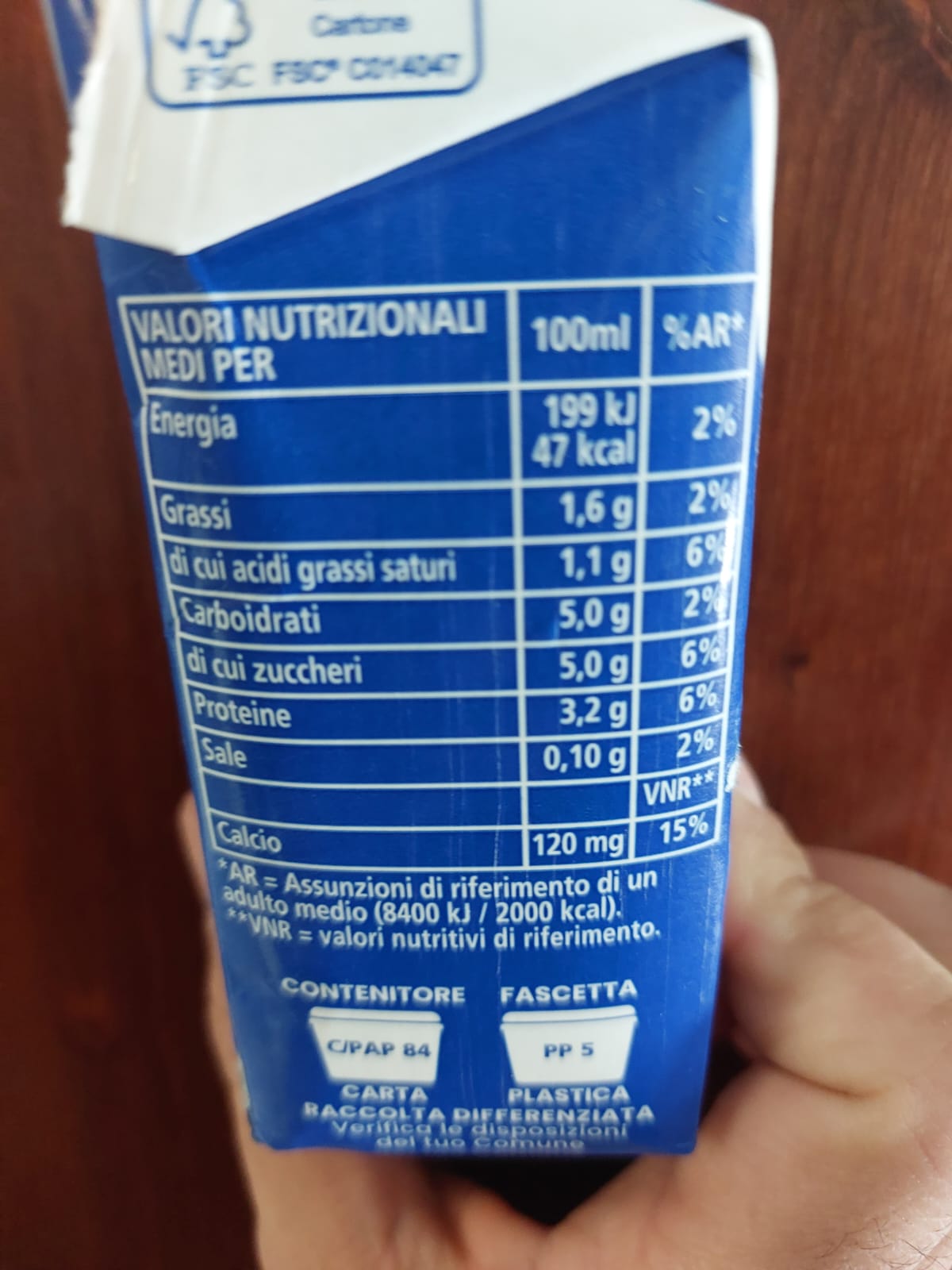
*

*Figure S11. Label of Milk*

*
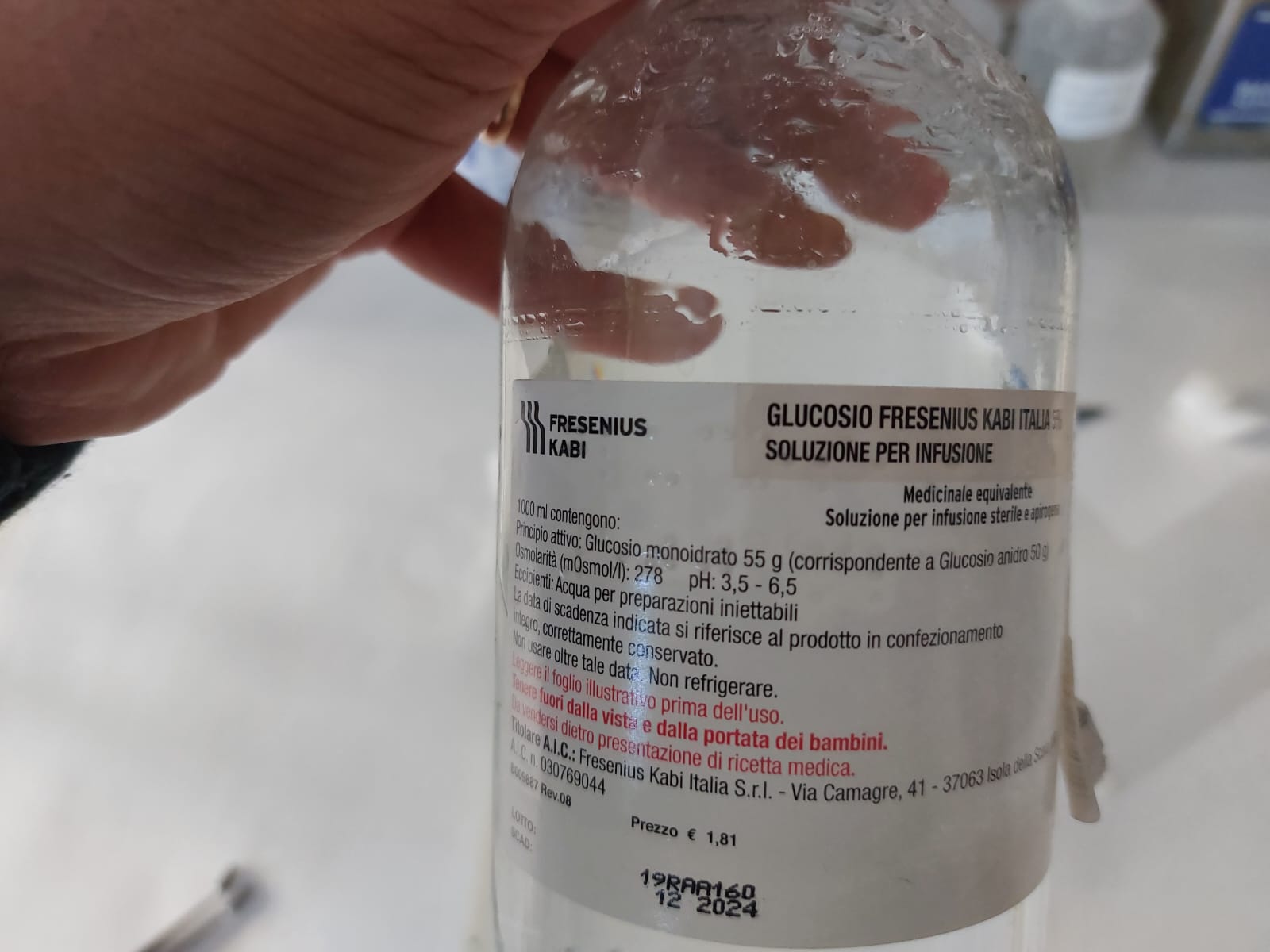
*

*Figure S12. Label of Isotonic solution*

**References**

[1] X. Zhu, C. Tang, H.-F. Wang, Q. Zhang, C. Yang, F. Wei, Dual-sized NiFe layered double hydroxides in situ grown on oxygen-decorated self-dispersal nanocarbon as enhanced water oxidation catalysts, J. Mater. Chem. A 3 (2015) 24540–24546. https://doi.org/10.1039/C5TA08019C.

[2] C. Li, Y. Su, S. Zhang, X. Lv, H. Xia, Y. Wang, An improved sensitivity nonenzymatic glucose biosensor based on a CuxO modified electrode, Biosensors and Bioelectronics 26 (2010) 903–907. https://doi.org/10.1016/j.bios.2010.07.007.

[3] Y. Ding, Y. Liu, J. Parisi, L. Zhang, Y. Lei, A novel NiO–Au hybrid nanobelts based sensor for sensitive and selective glucose detection, Biosensors and Bioelectronics 28 (2011) 393–398. https://doi.org/10.1016/j.bios.2011.07.054.

[4] D. Lakhdari, A. Guittoum, N. Benbrahim, O. Belgherbi, M. Berkani, Y. Vasseghian, N. Lakhdari, A novel non-enzymatic glucose sensor based on NiFe(NPs)–polyaniline hybrid materials, Food and Chemical Toxicology 151 (2021) 112099. https://doi.org/10.1016/j.fct.2021.112099.

[5] D. Basu, S. Basu, A study on direct glucose and fructose alkaline fuel cell, Electrochimica Acta 55 (2010) 5775–5779. https://doi.org/10.1016/j.electacta.2010.05.016.

[6] P. Parpot, S.G. Pires, A.P. Bettencourt, Electrocatalytic oxidation of d-galactose in alkaline medium, Journal of Electroanalytical Chemistry 566 (2004) 401–408. https://doi.org/10.1016/j.jelechem.2003.11.053.

[7] H. Druliolle, K.B. Kokoh, B. Beden, Electro-oxidation of lactose on platinum and on modified platinum electrodes in alkaline medium, Electrochimica Acta 39 (1994) 2577–2584. https://doi.org/10.1016/0013-4686(94)00257-6.

[8] J.W.E. Glattfeld, M.T. Hanke, THE OXIDATION OF MALTOSE IN ALKALINE SOLUTION BY HYDROGEN PEROXIDE AND BY AIR. THE PREPARATION AND STUDY OF MALTOBIONIC ACID., J. Am. Chem. Soc. 40 (1918) 973–992. https://doi.org/10.1021/ja02239a014.
